# Late mandibular fracture after third molar extraction: a malpractice case or not?

**DOI:** 10.5281/zenodo.15564456

**Published:** 2025-08-01

**Authors:** Sara Bernardi, Eleonora Ricciuti, Sara Trichilo, Davide Gerardi, Fabiola Rinaldi, Giuseppe Varvara, Stefano Mummolo, Guido Macchiarelli, Serena Bianchi

**Affiliations:** ^1^Department of Life, Health and Environmental Sciences, University of L’Aquila, L’Aquila, Italy. ^2^Department of Innovative Technologies in Medicine & Dentistry, Dental School, ‘G. D’Annunzio’ University of Chieti-Pescara, Chieti, Italy.

**Keywords:** Third molar extraction;, Mandibular fracture;, Forensic dentistry;, Pericoronitis

## Abstract

Fractures of the mandibular angle following surgical extraction of the third molar occur at an incidence ranging from 0.0034% to 0.0075%. The low incidence and the data present in the literature reveal how legal claims based on late mandibular fractures from third molar extractions are unlikely, being an uncommon clinical condition. The present case investigates the causal relationship between the fracture of the mandibular angle and the intervention of extraction of a dental element 3.8 in conditions of semi-inclusion and the possible hypothesis of dental malpractice. About two weeks after the extraction, the patient felt a noise like that produced by shattering glass, followed by severe and sudden pain along the area of the left mandibular joint and numbness. The following day, the patient underwent an orthopantomogram performed by the same medical team that carried out the operation in question, with an incorrect diagnosis of dislocation of the condyle, which is to be treated with muscle relaxants and anti-inflammatories. Upon further radiological investigations performed by different operators, it is concluded that the patient is suffering from a "fracture of the left mandibular angle”. The patient, therefore, reported and sued the dentists for the crime of negligent personal injury who had extracted element 3.8. From medical history, clinical examination, and documentation produced by the patient, it can be said that the extraction of element 3.8 was necessary as the pericoronary sac had caused an untreatable periodontal lesion at the distal root of the 3.7. From a medico-legal point of view, it was established that the extraction maneuvers may have caused the fracture of the mandibular angle, but it can be excluded professional responsibility in the criminal field of the medical team that carried out the res judicata intervention, since the fact in itself represents a known complication of the extraction of mandibular third molars.

## INTRODUCTION

The dental inclusions, specifically the third inferior molar or supernumerary teeth, are classified as eruptive disorders (*1*): during their extraction different complications could occur (*2*). The inclusion and semi-inclusion of the mandibular third molar are primarily due to the mandibular angle anatomy region, which is unable to provide sufficient space for tooth eruption (*3*). The surgical procedure for the extraction of impacted lower third molars can be associated with both intraoperative and postoperative complications, including severe pain, significant bleeding, extensive edema, potentially permanent inferior alveolar nerve injury, and even rare but serious mandibular fractures. (*4*, *5*) These complications, particularly in weak areas of the mandible such as the angle region, condylar region, and symphysis, underscore the gravity of the clinical situation. (*6*)

**Figure 1:**
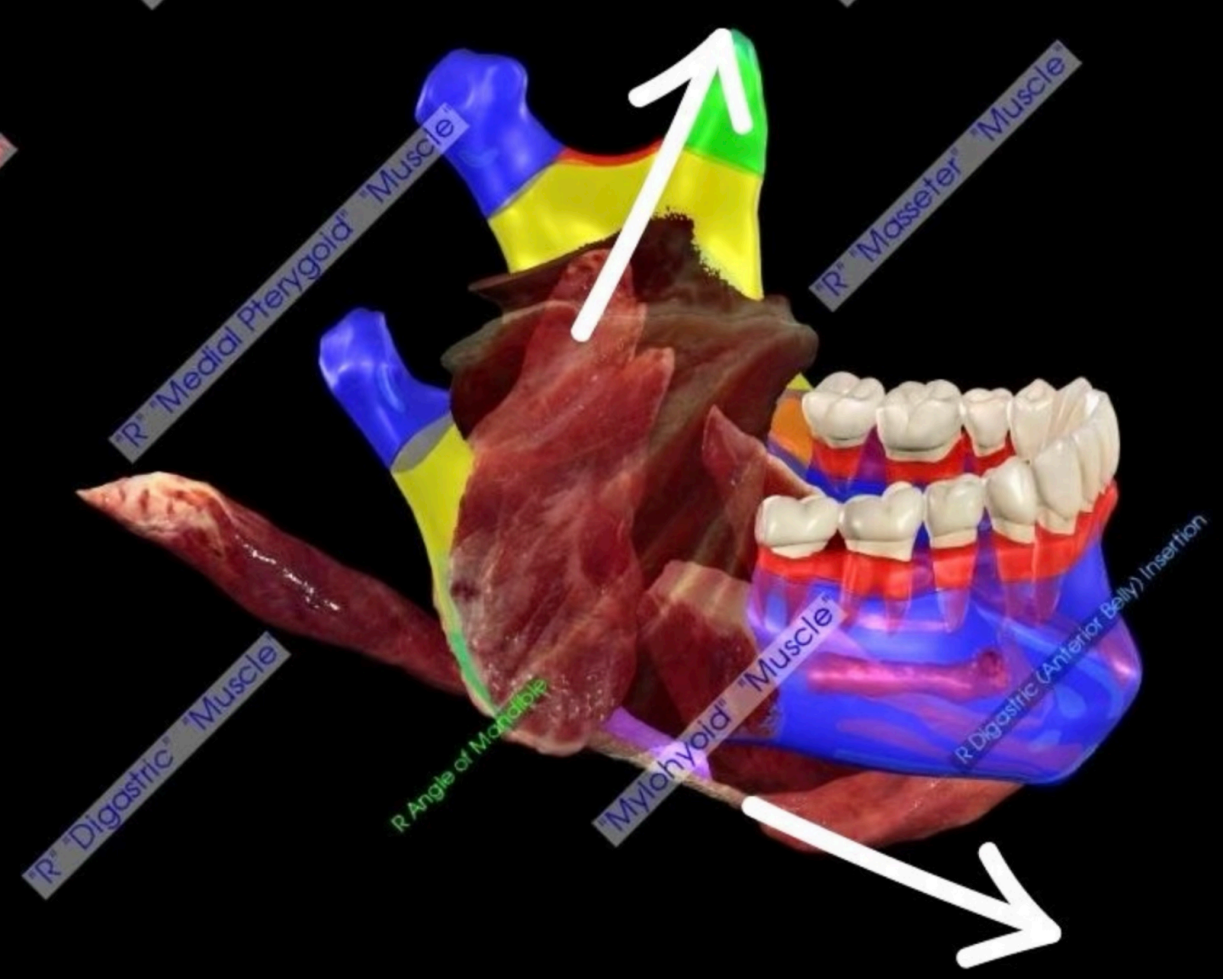
Anatomy of mandibular angle region with muscles inserted. Arrows show the vectorial forces developed during masticatory acts. Picture from Anatomage Inc. - Anatomage Table EDU. The 3D rendering of the donated body to science data is from Anatomage Table.

The mandibular angle is particularly vulnerable due to the transitional nature of the zone between the toothed and non-toothed portion of the mandible (*5*) and solicited by the vectorial forces of the masticatory and suprahyoid muscles (Figure 1).

The third molar, often partially impacted, reduces bone mass, making the structure more vulnerable and increasing the fracture risk. (*7*) Mandibular fractures associated with the extraction of lower third molars account for approximately 75% of all cases. (*5*) Factors contributing to the risk of fracture include the level of impaction on the surrounding bone, dental anatomy, characteristics of dental roots, previous infections, age, sex, postoperative period, bruxism, drugs affecting bone metabolism, and the patient's athletic activity. (*8*) Despite the relatively low incidence of this complication, ranging from 0.0033% to 0.0036% for intraoperative fractures and 0.0042% to 0.0046% for postoperative fractures (*9*), it's crucial for clinicians to be aware of the risks associated with retained impacted teeth, emphasizing the need for vigilance and caution in these procedures. It's important to note that the occurrence of late mandibular fractures, particularly those that become the subject of malpractice lawsuits, is a rarity in the literature. (*10*)

Given the low incidence, forensic dentistry associates the late mandibular fracture after third molar extraction as an unforeseen and unpredictable complication, provided the oral surgeon has diligently evaluated the medical history, excluded any factors predisposing to the fracture, informed the patient comprehensively, and used the hand-piece and surgical instruments correctly, while also monitoring the patient in the post-operative period. (*11*)

The law regulating medical responsibility in Italy is the 24/2017 law. The text of the law regulates the responsibility at the penal, civil, and administrative levels. Article 590 sexies of the Penal Code states, “When the lesion is due to incompetence, but the guidelines and literature indications are respected, liability is excluded”. (*12*) The penal liability of the health operator (doctor, dentist) is excluded for technical errors but not for negligence (when the operator does not take the appropriate precautions for the case) and for imprudence (when the operator does not respect guidelines). (*13*, *14*) In cases such as late mandibular fracture after third molar extraction, the operator should not be prosecuted criminally if the lesion is due to technical errors.

Here, we report a case of a late mandibular fracture after third molar extraction. The operators and their professional actions have been investigated by technical consultants of the prosecutor’s office to assess their eventual criminal responsibility.

## THE CASE

A 40-year-old male patient went to a private healthcare structure affiliated with an insurance company, reporting an episode of inflammation at the level of the left hemiarch of the mandible. After undergoing an orthopantomography (OPG) conducted by dentist “1”, the patient underwent extraction of tooth 3.8 by dentist “2”, operating in the same healthcare facility. The patient reported that the post-operative course was uneventful until seven days after the removal when, during lunch, he reported hearing a noise like “breaking glass,” followed by intense pain. He stated that he contacted the healthcare facility and went there for a check-up. Furthermore, the patient reported that, after examination of the X-ray, a dislocation of the condyle was diagnosed; therefore, the treatment prescribed was based on anti-inflammatory drugs and muscle relaxants, and another appointment for the following day was scheduled.

On the same evening, the patient reported that, after a discussion with his wife, it seemed advisable to seek a second opinion and, therefore, contacted Dentist 3, his wife’s physician. The next day, the patient went to Dentist 3’s office and underwent further examination with OPG and Computerized Tomography (CBCT): the radiographic exams revealed a fracture of the angle of the left mandible, and Dentist “3” decided to treat it through arch ligation for four weeks. After four weeks, the previous treatment was ineffective, and a new treatment consisting of immobilization with metallic wire and brackets was scheduled. Due to the above-described events, the patient made a formal complaint to Dentists “1” and ”2”, suing for personal injuries.

## Technical consultation operations of the public prosecutor

Following the complaint filed by the patient, the public prosecutor proceeded to appoint their consultants to ascertain:

§ Any responsibilities for fault, malpractice, and negligence of the current suspects;
§ The necessary precautions by the individuals and/or medical personnel who have treated the patient; in affirmative case, the consultants must individuate expressly the negligent conduct (commission or omission) and the person responsible for it, as well as representing, according to medical science evaluations, the causal relationship between the negligent conduct (active and omissive) and the diagnosed injuries listed below:
  - Circular bone lesion of large dimensions as a result of osteotomy in position 4.8 and still the presence of well-represented radicular fragments;
  - Circular bone lesion of large dimensions as a result of osteotomy in position 3.8;
  - Fracture of the angle of the left mandible
§ The severity of the injuries suffered by the victim, including the relation to the days of prognosis, as direct consequences of any negligent or intentional conduct attributable to the suspects.

The Technical Consultation Operations were performed in the presence of the prosecutor's office's panel of technical consultants, the party’s technical consultants, and the patient. After identifying the expert witness, anamnestic data were collected. The medical history resulted clear and without any peculiarities to report.

He reported taking painkillers three times a day in the period following the events in question and that he had taken psychotropic drugs for sleep until September 2021.

## Extraoral and intraoral examinations.

At the time of the expert operations, the patient reported a dull-discontinuous pain associated with a level 5 on the Visual Analogue Scale (VAS).

**Figure 2:**
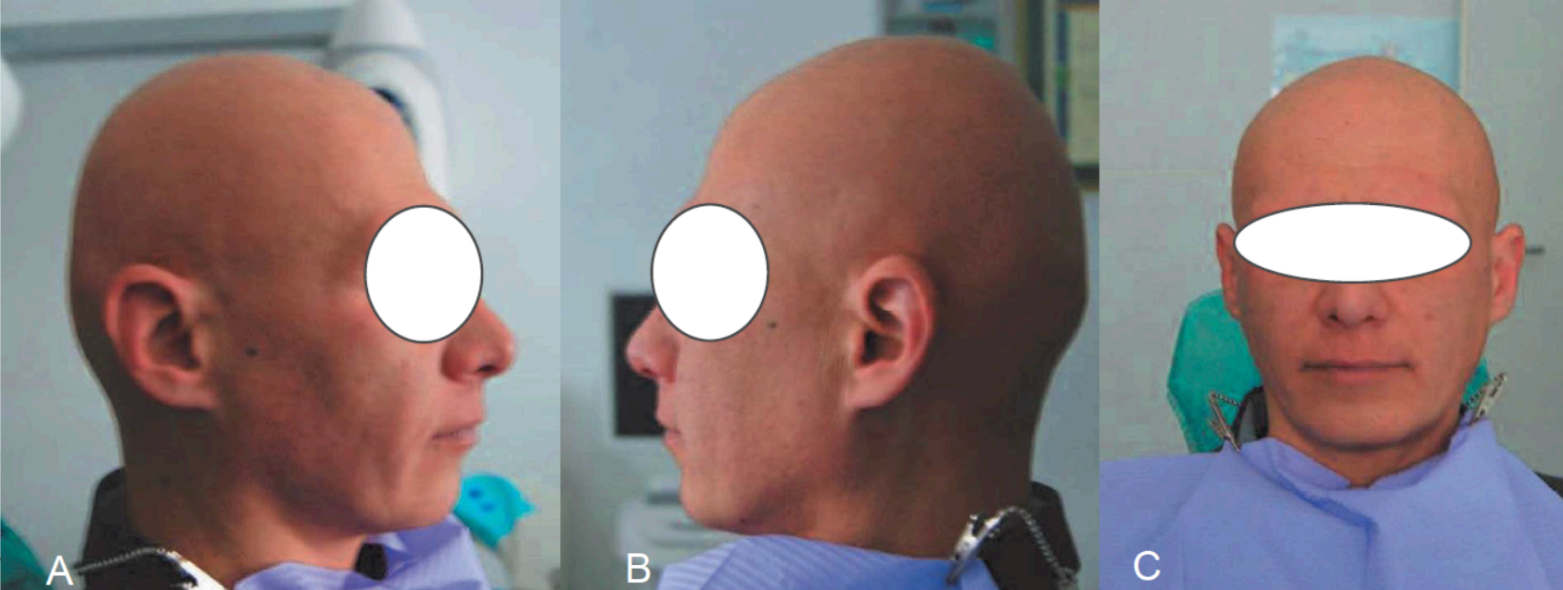
a.b.c. Extraoral images showing the absence of visible asymmetries

**Figure 3:**
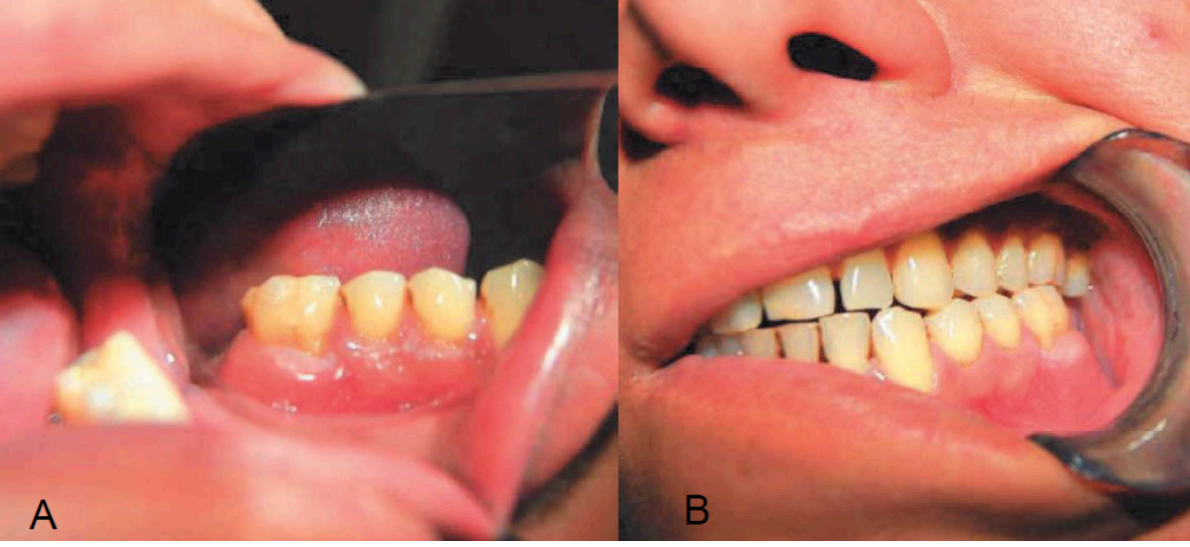
a.b. Intraoral images showing an area of resorption of the left alveolar ridge

**Figure 4:**
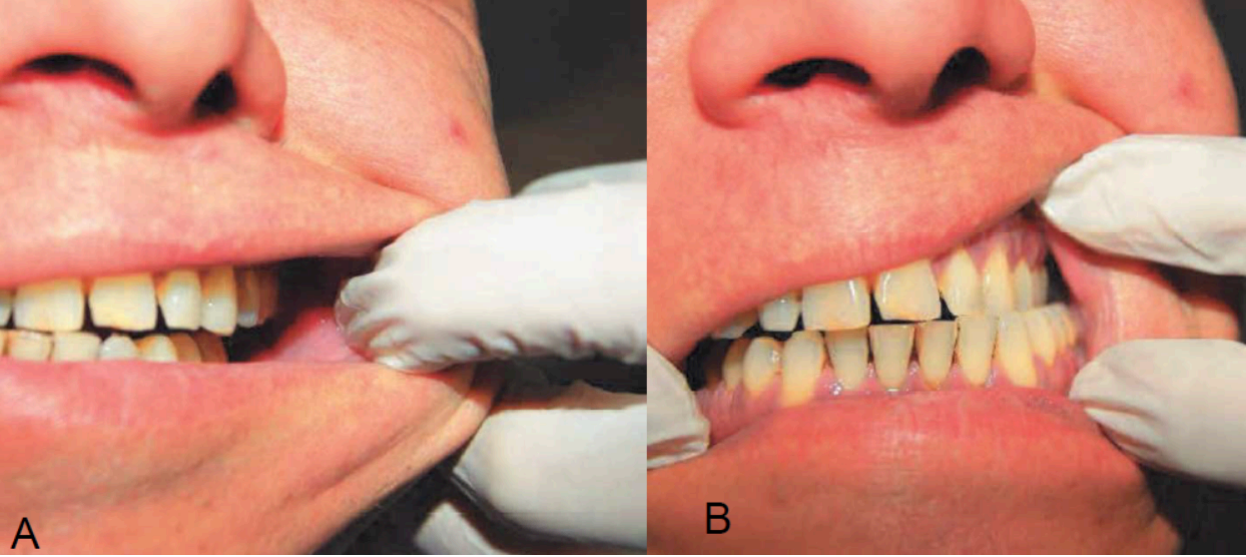
a.b. Examination of the lateralization function appearing congruent and functional

No asymmetries were appreciable (Fig. 2a, b, and 2c). Intraorally, an alveolar crestal bone resorption is present (Figure 3a, 3b), and mandibular dynamics appear congruent and functional (Fig. 4a, 4b).

## Examination of the radiograms.

**Figure 5:**
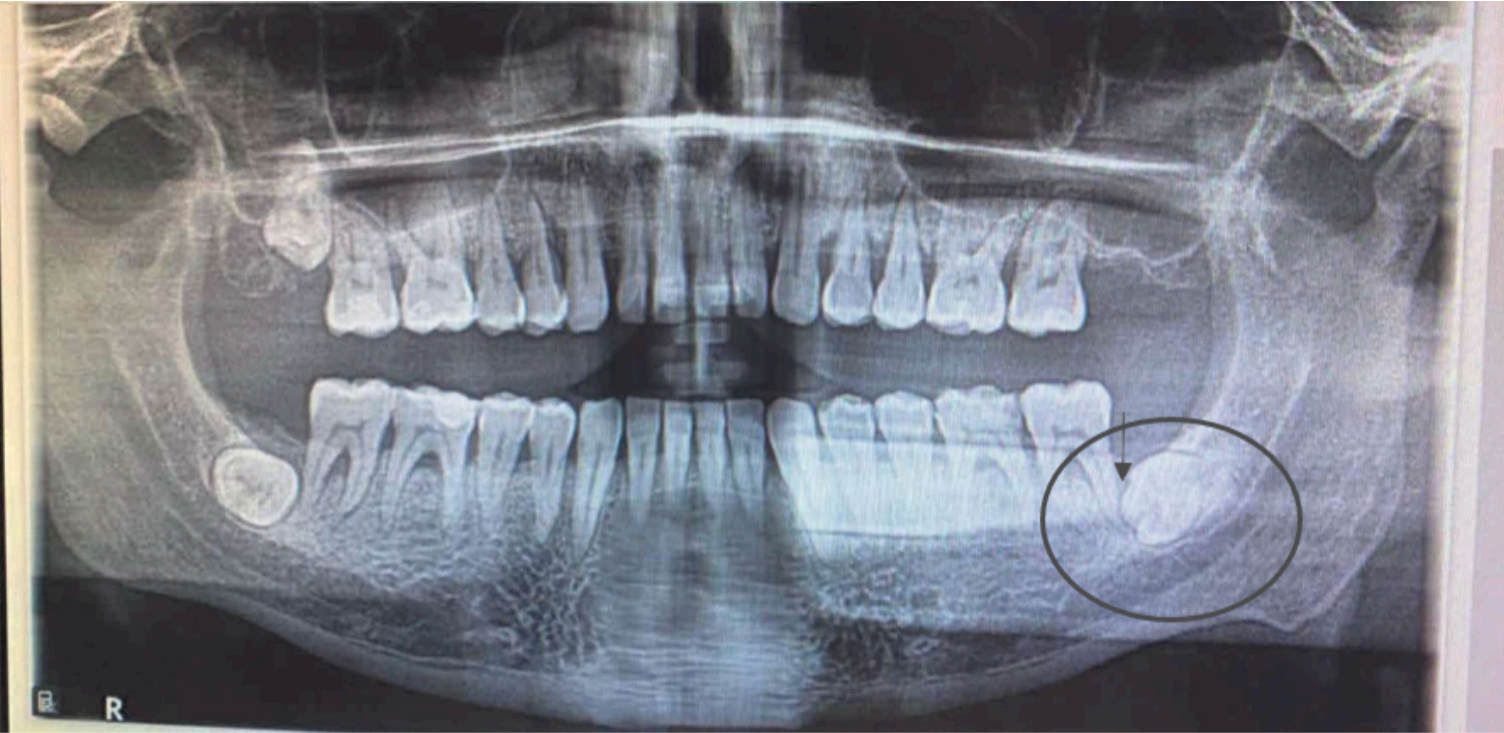
Pre operative OPG. The circle and the arrow indicated the 3.8 tooth

The pre-operative OPG (Figure 5) showed the presence of the left inferior third molar in a state of semi-inclusion, class IB according to the Pell & Gregory classification. The pericoronal sac shows mesially to have caused resorption of the supporting bone tissue at the distal root of the seventh.

**Figure 6:**
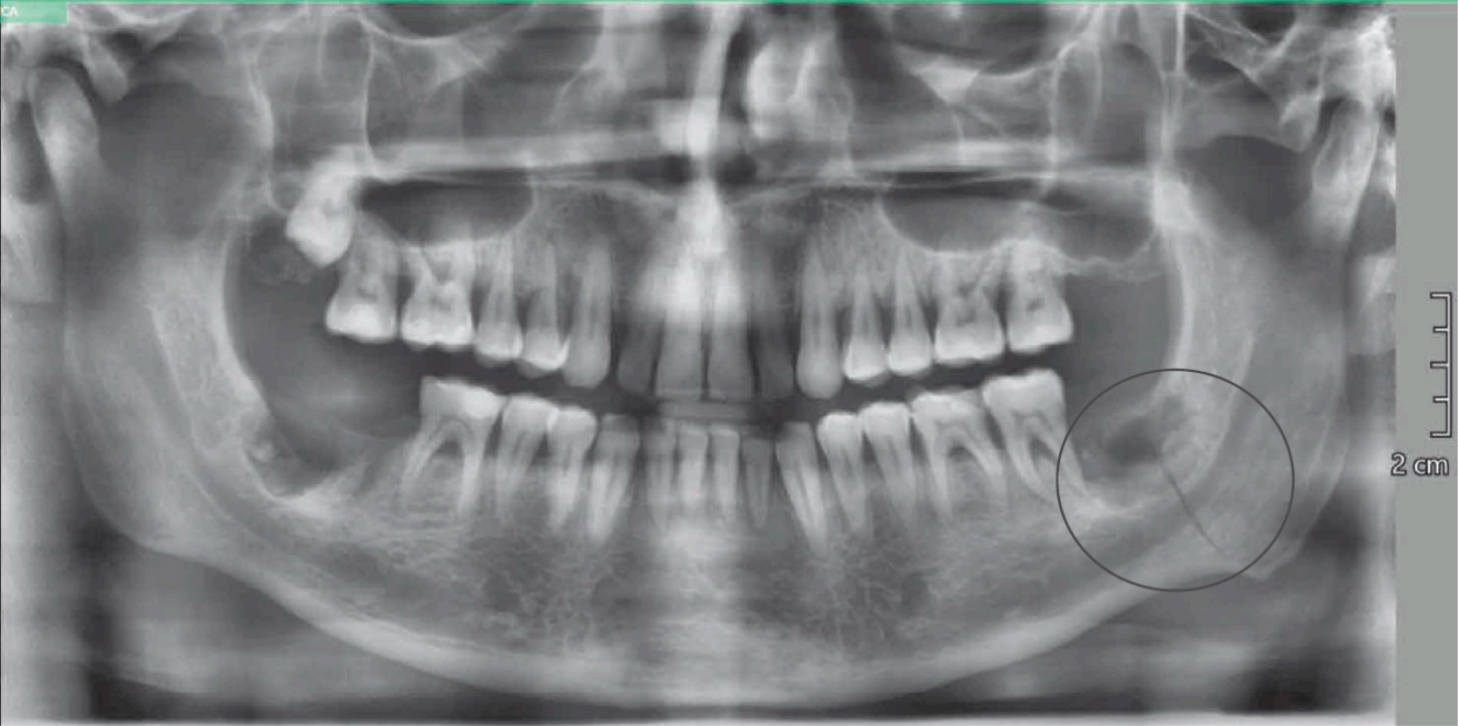
OPG performed after the onset of the post-operative symptoms. The circle indicated the fracture line

The OPG performed the day of onset of the symptoms showed a lack of continuity at the posterior border of the left mandible (Figure 6).

**Figure 7:**
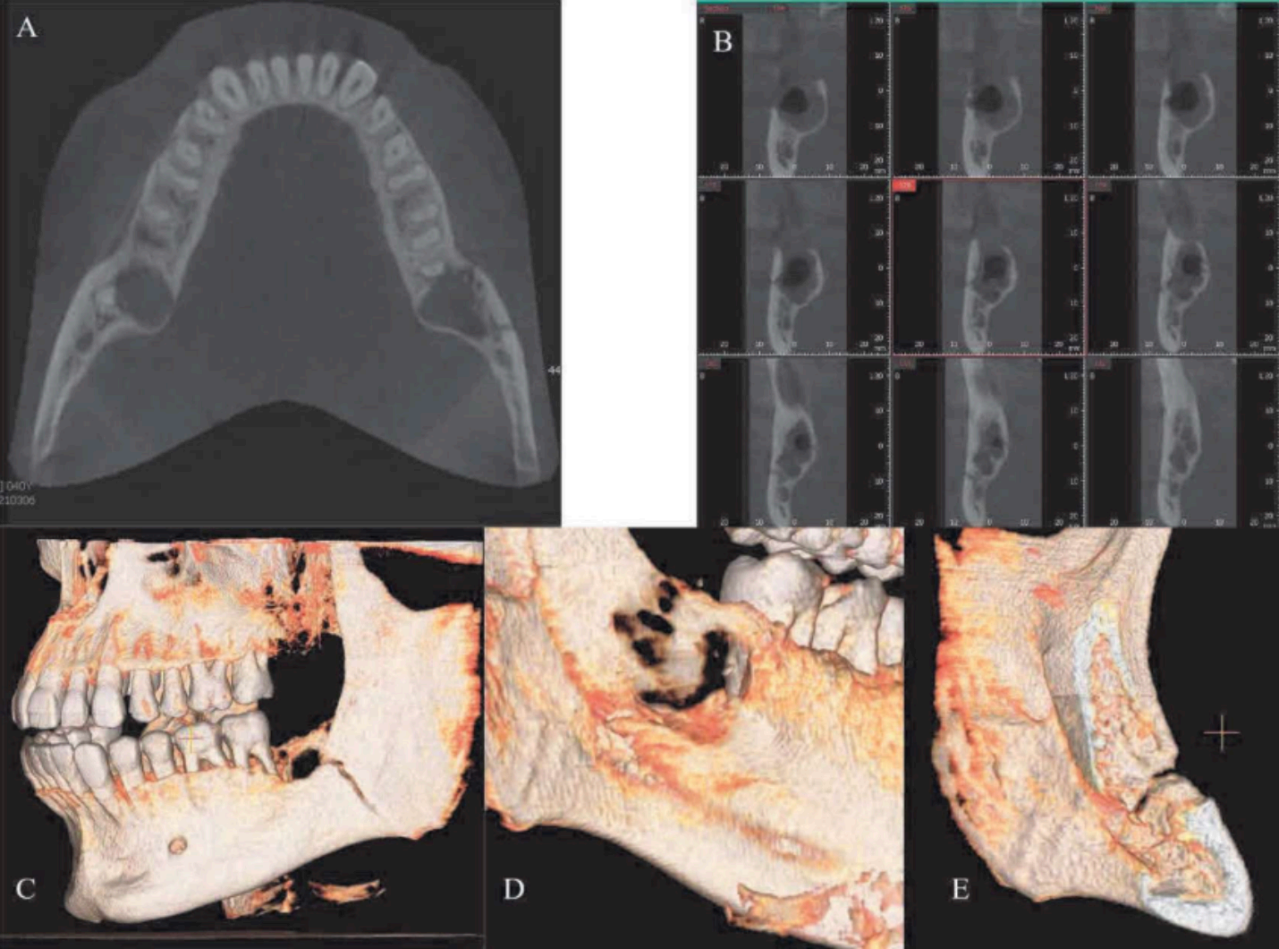
A) Axial projection where it is possible to appreciate the fracture line crossing the cortical bone sharply (arrow). B) Cross-sections: the fracture line crosses the cortical bone sharply and extends through multiple sections. C)-D)-E): 3D reconstruction showing how the fracture is identified as an incomplete/nearly complete type, given the small amount of residual bone not involved at the level of the mandibular border. The classification of this fracture is therefore on the borderline between an incomplete and complete fracture type with minimal displacement of the fragments

The CBCT showed the presence of the lack of continuity of the angle of the left mandible involving the lingual side as well (Figure 7).

the fracture is identified as an incomplete/nearly complete type, given the small amount of residual bone not involved at the level of the mandibular border. The classification of this fracture is therefore on the borderline between an incomplete and complete fracture type with minimal displacement of the fragments.

**Figure 8:**
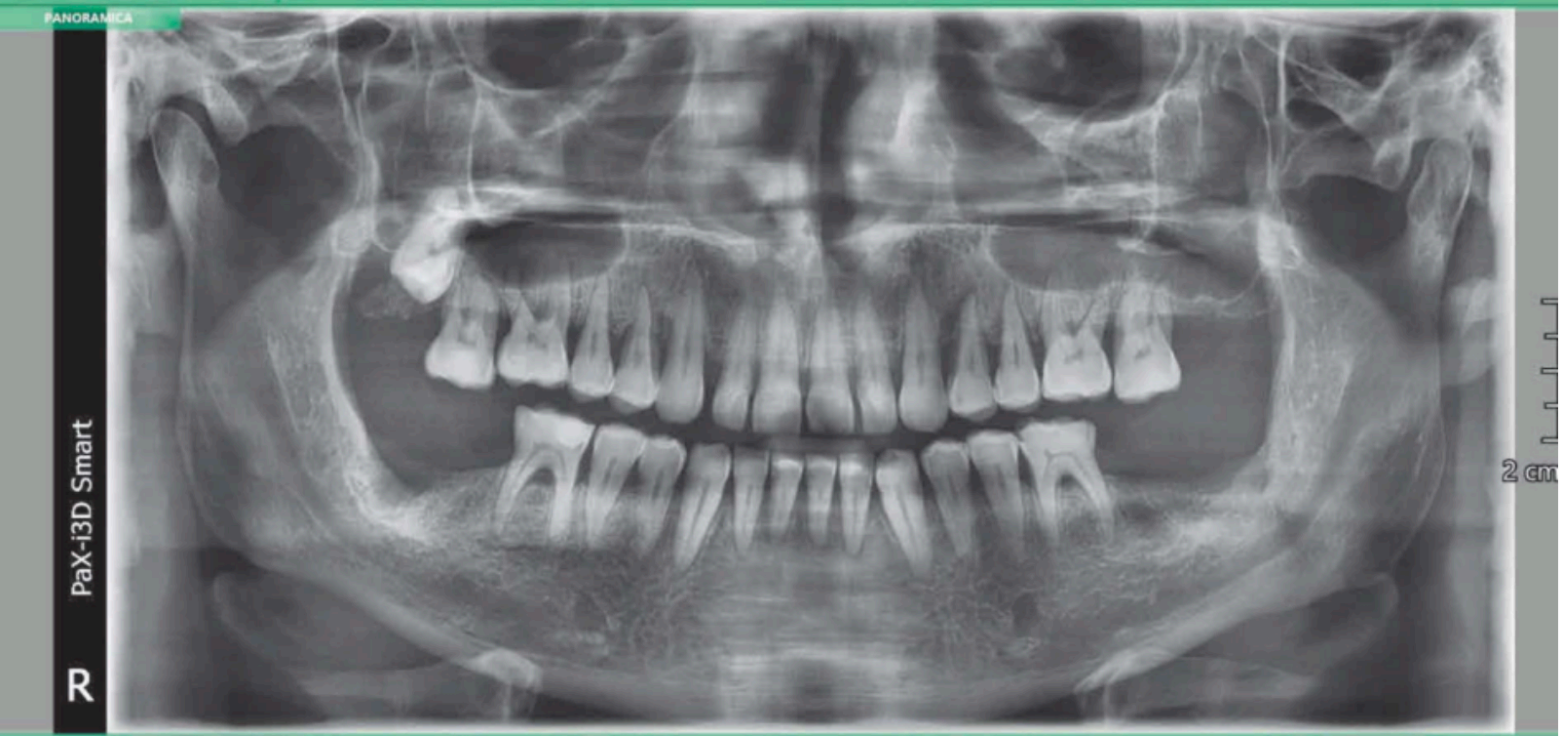
OPG carried out after two months after the treatment of the fracture

The OPG carried out more than two months later showed that the healing process of the fracture line is underway. The apices of the 4.7 are still within the bone (Figure 8).

## Medico-legal considerations

From the medical history, the clinical examination, and the documentation produced, as well as from the results of the new instrumental tests carried out during the appraisal, it is possible to state that:

1.The services provided by dentist 2 included the extraction of element 3.8, with the appearance of a fracture of the angle of the mandible after two weeks. As regards the services provided by dentist 1, they did not produce criminally relevant injuries to the patient;
2.From the examination of the pre-operative OPG, the extraction of the 3.8 was indicated as the pericoronary sac at the mesial level, showing that it has caused an untreatable periodontal lesion at the level of the distal root of the seventh. The prevention of infectious pathologies and the presence of signs of periodontal pathology are among the indications for extraction, according to the Italian Clinical Recommendations in Odonto-stomatology ed. 2017 (*15*);
  1. The fracture of the angle, although it occurred unusually, i.e., without any prodromal and opening symptoms, is a complication of the extraction of the semi-impacted lower third molar which, although rare, can occur after the extraction (*16*);
4.The osteotomy results detected radiographically at sites 3.8 and 4.8 are compatible with the osteotomy maneuvers necessary for the extraction of impacted third molars (*16*);
5.As regards the roots remaining from 4.7, and still present at the OGT of the last follow-up, produced during the appraisal, given that these did not give inflammatory results, it does not constitute a harmful act.

Considering the second intervention by dentist 3, not conclusive, as the fracture did not appear to be a greenstick fracture but an actual fracture of the angle which would have required immediate intervention by a specialist in maxillofacial surgery, with block rigid intermaxillary or application of intermaxillary fixation screws (IMF) between the third and fourth tooth to allow a stable intermaxillary block and reduction of operating times (*17*), it was verified that the interruption of the causal link between the injury and the state of health suffered by the injured person and objectified during this medico-legal investigation.

Ultimately, the medico-legal analysis allows us to state that the extraction maneuvers may have caused the fracture of the mandibular angle, but that this event appears to be among the complications of the lower third molar extraction operation (*8*). Since dentist 2 performed the operation according to clinical recommendations and good practices, it does not constitute criminally relevant personal injury.

## DISCUSSION

### The late mandibular fracture after third molar removal: a remote possibility or concrete probability

The case presented offers a unique perspective on a relatively rare complication that can arise following a frequent surgical procedure. The incidence of mandibular fracture after third molar extraction (late fracture) is indeed rare, occurring in less than 1% of cases.^(18-20)^ This rarity underscores the importance of understanding and managing this potential complication. In the literature, most authors report the onset of late fractures between the second and fourth week after surgical extractions, during chewing, especially during meals (*18*). It has also been reported that male patients over 40 have a higher incidence of late mandibular fractures.^(19-21)^ In the case presented, the fracture seems to have occurred in the abduction phase of the mandible. Usually, the fracture is associated with an atrophic mandible, an impacted lower third molar, a large tooth volume (associated or not with bone cysts or tumors) (*19*, *22*), poor surgical planning, poor technique (adequate, inadequate instrumentation, and excessive use of manual force instruments during the extraction procedure). In the case presented, from the clinical documentation presented, there is no evidence of an inadequate use of instruments or surgical instruments unsuitable for the operations.

A combination of forces from the masticatory muscles (masseter, medial pterygoid, and digastric) on a possible fragile jaw can increase the incidence of fractures. (*19*-*21*, *23*-*25*) Further causes, such as systemic disorders that cause bone fragility (osteoporosis, hyperparathyroidism, rheumatism, osteogenesis imperfecta, and Pajet's disease), and local trauma with impact on the surgical site can lead to late mandibular fractures. (*20*-*22*, *24*) During the extraction, it is crucial to remember that meticulous surgical planning is always preferable, including performing as many dental sections as necessary, minimizing the osteotomy, and, consequently, reducing the fragility of the jaw and the incidence of fractures. (*19*, *26*, *27*) It is described in the literature that the use of the piezoelectric handpiece for osteotomy is indicated to re-duce excessive forces during tooth avulsion and reduce manipulation of neural structures, avoiding paresthesia. (*28*) Faced with the potential incidence of late mandibular fractures, patients must be informed to maintain a soft diet for at least 4 weeks. (*25*)

Another factor that predisposes to fractures is the position of the dental element and the side where it is positioned. Although the distoangular position is generally considered the most technically difficult compared to the others, it requires more extensive bone removal. In the study of Galvao et al., however, the mesioangular and vertical angulations have been associated with the highest incidence of fractures despite being the most accessible positions to operate on and requiring less bone removal. (*29*) In terms of tooth position, class II and III, B, and C cases were found to have a higher incidence of mandibular fracture than class I and A cases. (*29*) This is probably linked to a greater degree of extraction difficulty and removal of more extensive bone. There was also a higher incidence of mandibular fracture for ultimately impacted teeth (64.8%) than partially impacted teeth. (*29*) When the tooth is completely covered by bone, it typically occupies a more significant portion of the mandibular angle and requires more bone removal during surgery. Post-operatively, this leads to a smaller amount of residual cortical bone and, therefore, a more fragile mandibular angle, which can be a significant causal factor of late fracture. (*8*) It is crucial to educate patients about these potential risks and the importance of post-operative care, including maintaining a soft diet for at least 4 weeks, to minimize the incidence of late mandibular fractures.

As regards the hemimandible, the left side represents a possible risk factor that increases the incidence of fracture.

In the study by Wagner et al. (2005), teeth located on the left side of the mandible may require a major osteotomy due to the difficulty of visualization found in most right-handed operators. (*30*)

In cases where an extensive osteotomy is necessary, it is prudent to administer the patient a bland liquid diet, especially during the first four weeks. This is the most critical period, when there is a greater probability of post-operative mandibular fractures. (*31*)

In the case presented, in addition to being a 40-year-old male patient, the fracture occurred on the left side, in agreement with the few data in the literature

### The forensic dentistry point of view.

The case, beyond to be a fortuitous finding of a rare complication of third molar surgery, is the subject of medical-legal litigation in the criminal field. Specifically, the errors which are the subject of the complaint and attributed to dentists 1 and 2 by the party's consultants are two: errors of a technical nature (not verifiable with absolute certainty from the clinical and radiographic documentation produced) and of a diagnostic omission type (non-radiographic diagnosis of the fracture). The medico-legal analysis of the documentation produced found compliance with the clinical recommendations in dentistry in the indication for the extraction of the lower third molar and could only assume but not as-certain a causal connection between a technical error and the harmful event. The failure to diagnose the fracture is also a technical error: upon reporting the symptoms, dentist 2 promptly carried out the radiographic examination as well as wanted to check the patient again the following day. The intervention of dentist 3 interrupted the causal link between the incorrect diagnosis and the state of illness, prolonging and worsening it. However, what can also be seen from the immediate change of doctor by the patient is a significant lack of attention on the part of the suspects towards correct information for the patient. The "hardest" litigation is not based exclusively on technical errors and the lack of clinical documentation demonstrating that all the best means available in the specific case were used, but on the lack of communication; every single intervention will require adequate support, calibrating this support on the type of intervention and its complexity, as well as on the type of patient and his need to “know". (*32*) In this specific case, the patient reported that he had not been informed of this possible complication, although rare, nor had he received any prescriptions regarding its prevention.

The incorrect diagnosis that subsequently occurred severely damaged the doctor-patient relationship of trust, pushing the plaintiff first to change doctor within a few hours and subsequently to turn to criminal rather than civil law. The late onset of post-surgical mandibular fracture appears to be relatively rare, and the potential risks inherent to this com-plication, in the case of subjects with an unfavorable medical history for systemic bone pathologies, are often not explained to the patient. This case highlights the crucial role of active patient information in daily clinical practice, especially in cases of complex surgical interventions. Informed consent and a dialogue duly suited to the patient's level of under-standing play a significant role in this scenario as a tool for clinical communication, self-awareness, guarantee of the right to self-determination, therapeutic alliance, and consolidation of the doctor-patient relationship of trust.

## CONCLUSION

If the mandibular third molars need to be removed, the oral surgeon should carefully evaluate the patient before performing the surgery, use appropriate instruments to minimize any potential complications, and spend enough time with the patient to explain the risks and complications that may arise after the surgery. One of the rarest post-operative complications is the fracture of the mandibular angle.

## References

[1] Bailey E Kashbour W Shah N Worthington HV Renton TF Coulthard P. Surgical techniques for the removal of mandibular wisdom teeth. Cochrane Database Syst Rev. 2020;7:CD004345. 10.1002/14651858.CD004345.pub3 32712962 PMC7389870

[2] Varvara G Angiolani F Rinaldi F Gerardi G Bernardi S Rastelli S Complication of the extraction of maxillary anterior supernumerary teet: the accidental extraction of the permanent tooth bud. Bull Stomatol Maxillofac Surg. 2025;21:29–35. 10.58240/1829006X-2025.1-29

[3] Wang D He X Wang Y Zhou G Sun C Yang L Topographic relationship between root apex of mesially and horizontally impacted mandibular third molar and lingual plate: cross-sectional analysis using CBCT. Sci Rep. 2016;6:39268. 10.1038/srep39268 27991572 PMC5171861

[4] Varvara G Bernardi S Piattelli M Cutilli T. Rare and life-threatening complication after an attempted lower third molar extraction: Lemierre syndrome. Ann R Coll Surg Engl. 2019;101:e52–4. 10.1308/rcsann.2018.0190 30372118 PMC6351862

[5] Mottl R Kunderová M Slezák R Schmidt J. Iatrogenic Fracture of the Lower Jaw: A Rare Complication of Lower Molar Extraction. Acta Medica (Hradec Kralove). 2021;64:101–7. 10.14712/18059694.2021.18 34331430

[6] Shivani N Senthil Murugan P Leelavathi L. Correlation of mandibular 3rd molars with angle fractures. Int J Dent Oral Sci. 2021;8:1460–5.

[7] Palmela Pereira C Santos R Santos A Gonçalves C Augusto D Rodrigues A A systematic review and meta-analysis of oral and maxillofacial trauma. J Forensic Odontostomatol. 2022;40:2–21. 36623294 PMC10266705

[8] Pires WR Bonardi JP Faverani LP Momesso GA Muñoz XM Silva AF Late mandibular fracture occurring in the postoperative period after third molar removal: systematic review and analysis of 124 cases. Int J Oral Maxillofac Surg. 2017;46:46–53. 10.1016/j.ijom.2016.09.003 27688170

[9] Guillaumet-Claure MA Juiz-Camps AM Gay-Escoda C. Prevalence of intraoperative and postoperative iatrogenic mandibular fractures after lower third molar extraction: A systematic review. J Clin Exp Dent. 2022;14:e85–94. 10.4317/jced.58390 35070129 PMC8760961

[10] Hupp JR. Legal implications of third molar removal. Oral Maxillofac Surg Clin North Am. 2007;19:129–36, viii. . 10.1016/j.coms.2006.11.008 18088871

[11] Giovacchini F Paradiso D Bensi C Belli S Lomurno G Tullio A. Association between third molar and mandibular angle fracture: A systematic review and meta-analysis. J Craniomaxillofac Surg. 2018;46:558–65. 10.1016/j.jcms.2017.12.011 29459187

[12] Cupelli C. La responsabilita penale degli operatori sanitari e le incerte novità della legge Gelli-Bianco. Cassaz. Penal. 2017, 1765–1778.

[13] Scarpelli ML, Pinchi V, Fiore C. L’odontoiatria e la responsabilità professionale (dopo la legge Gelli/Bianco); Ariesdue, 2018; ISBN 8898789130.

[14] Corte-Real A Caetano C Dias Pereira A Rocha S Alves S Nuno-Vieira D. Risk and limits in dental practice: a Portuguese approach to medical-legal evaluation and professional liability. J Forensic Odontostomatol. 2020;38:2–7. 32420907 PMC7880157

[15] Gherlone E, Allegrini S, Annibali S, Baggi L, Barbato E, Barone A, Berutti E, Braga G, Branchi R, Brenna F, Caiazzo A. Raccomandazioni cliniche in odontostomatologia. 2017.

[16] Chiapasco M. Manuale illustrato di chirurgia orale-IV edizione.; Edra, 2020.

[17] Baldisserri E, Bassi M, Benech A, Berrone S, Bertossi D, Bianchi A, et al. Trattato di Patologia Chirurgica Maxillo-Facciale. In Trattato di Patologia Chirurgica Maxillo-Facciale; minerva medica, 2007 ISBN 8877115661.

[18] Delantoni A Antoniades I. The iatrogenic fracture of the coronoid process of the mandible. A review of the literature and case presentation. Cranio. 2010;28:200–4. 10.1179/crn.2010.028 20806739

[19] Chrcanovic BR Custódio AL. Considerations of mandibular angle fractures during and after surgery for removal of third molars: a review of the literature. Oral Maxillofac Surg. 2010;14:71–80. 10.1007/s10006-009-0201-5 20091416

[20] Azenha MR Kato RB Bueno RB Neto PJ Ribeiro MC. Accidents and complications associated to third molar surgeries performed by dentistry students. Oral Maxillofac Surg. 2014;18:459–64. 10.1007/s10006-013-0439-9 24370576

[21] Grau-Manclús V Gargallo-Albiol J Almendros-Marqués N Gay-Escoda C. Mandibular fractures related to the surgical extraction of impacted lower third molars: a report of 11 cases. J Oral Maxillofac Surg. 2011;69:1286–90. 10.1016/j.joms.2010.05.059 21193255

[22] Kao YH Huang IY Chen CM Wu CW Hsu KJ Chen CM. Late mandibular fracture after lower third molar extraction in a patient with Stafne bone cavity: a case report. J Oral Maxillofac Surg. 2010;68:1698–700. 10.1016/j.joms.2009.06.019 19939535

[23] Giovannetti F Lupi E Di Giorgio D Scarsella S Oliva A Di Fabio D Impact of COVID19 on Maxillofacial Fractures in the Province of L’Aquila, Abruzzo, Italy. Review of 296 Patients Treated With Statistical Comparison of the Two-Year Pre-COVID19 and COVID19. J Craniofac Surg. 2022;33(4):1182–4. 10.1097/SCS.0000000000008468 36041111 PMC9232240

[24] Novelli G Sconza C Ardito E Bozzetti A. Surgical treatment of the atrophic mandibular fractures by locked plates systems: our experience and a literature review. Craniomaxillofac Trauma Reconstr. 2012 June;5(2):65–74. 10.1055/s-0031-1300961 23730420 PMC3444021

[25] Al-Belasy FA Tozoglu S Ertas U. Mastication and late mandibular fracture after surgery of impacted third molars associated with no gross pathology. J Oral Maxillofac Surg. 2009;67(4):856–61. 10.1016/j.joms.2008.09.007 19304046

[26] Bodner L Brennan PA McLeod NM. Characteristics of iatrogenic mandibular fractures associated with tooth removal: review and analysis of 189 cases. Br J Oral Maxillofac Surg. 2011;49(7):567–72. 10.1016/j.bjoms.2010.09.007 20947226

[27] Pippi R Solidani M Broglia S Cristalli MP. Prevention of mandibular fractures caused by difficult surgical extractions: report of a borderline case. J Oral Maxillofac Surg. 2010;68(5):1162–5. 10.1016/j.joms.2009.07.054 20188450

[28] Noetzel N Fienitz T Kreppel M Zirk M Safi AF Rothamel D. Osteotomy speed, heat development, and bone structure influence by various piezoelectric systems-an in vitro study. Clin Oral Investig. 2019;23(11):4029–41. 10.1007/s00784-019-02838-8 30826919

[29] Galvão EL da Silveira EM de Oliveira ES da Cruz TMM Flecha OD Falci SGM Association between mandibular third molar position and the occurrence of pericoronitis: A systematic review and meta-analysis. Arch Oral Biol. 2019;107:104486. 10.1016/j.archoralbio.2019.104486 31374491

[30] Wagner KW Otten JE Schoen R Schmelzeisen R. Pathological mandibular fractures following third molar removal. Int J Oral Maxillofac Surg. 2005;34(7):722–6. 10.1016/j.ijom.2005.03.003 15878820

[31] Duarte BG Assis D Ribeiro-Júnior P Gonçales ES. Does the Relationship between Retained Mandibular Third Molar and Mandibular Angle Fracture Exist? An Assessment of Three Possible Causes. Craniomaxillofac Trauma Reconstr. 2012;5(3):127–36. 10.1055/s-0032-1313355 23997857 PMC3578651

[32] Esposito N, Ferro S, Manchisi M, Mirenghi S, Paganelli P, Scarpelli ML. Prevenire e gestire il contenzioso in caso di insuccesso clinico; Quaderni ANDI Assicura, OrisBroker. 2015.

